# Social calls of *Myotis nattereri* during swarming: Call structure mirrors the different behavioral context

**DOI:** 10.1371/journal.pone.0221792

**Published:** 2019-09-06

**Authors:** Philipp Schmidbauer, Annette Denzinger

**Affiliations:** Animal Physiology, Institute for Neurobiology, University of Tübingen, Tübingen, Germany; McGill University, CANADA

## Abstract

Swarming is a characteristic behavior of bats that occurs in different social contexts. We studied the swarming behavior of *Myotis nattereri* at a maternity colony and at an autumn swarming site in South-West Germany by using synchronized sound and video recordings. Swarming was always associated with social vocalizations consisting of four frequently occurring call types. Call type A was a short call with a broadband steep-shallow-steep downward frequency modulation. Call type B consisted of two elements beginning with a broadband upward hooked element followed by a steep frequency modulated element. Call type C showed a characteristic rapid downward-upward-downward frequency modulation. Call type D was a long sinusoidal trill-like call with high variability in signal structure. All call types were recorded at the maternity colony, as well as at the autumn swarming site, but the incidence of each call type differed distinctly between the study sites. At the maternity roost, type A calls were most commonly produced. We found evidence for an individual signature in this call type and suggest that this social call has the function of a contact call in Natterer’s bats. At the autumn swarming site, type D calls were the most common social calls; in contrast, this call type was recorded only twice at the maternity roost. The occurrence of trills mainly at the autumn swarming site and their high variability suggests that trills function as male advertisement calls in *M*. *nattereri*.

## Introduction

Many bat species live in groups with complex social structures where communication with conspecifics plays a crucial role [1]. Like in many other animal taxa, communication in bats can be mediated by acoustic, olfactory, visual, and tactile signals [2]. Acoustic communication is the most suitable modality for social communication for highly mobile, nocturnal animals like bats [2, 3]. Vocalizations are independent from illumination and transfer information over long distances. As an adaptation to long distance transmission social calls are usually lower in frequency and show a higher complexity than echolocation calls [3–5]. Social calls that are produced by bats during flight can be differentiated into three groups based on their function and signal structure. Social integration calls, that facilitate recognition and often encode individual signatures, usually show a variable frequency modulated signal shape. Conflict resolution signals, that are produced in disagreement over limited resources, are noisy and low in frequency when they are produced in roosts, while in-flight conflict resolution calls show a buzz-like signal structure. Courtship vocalizations, that are used to attract mating partners or defend them against rivals, are often complex and composed of several diverse elements [5]. In bats, echolocation calls also contain social information such as the sex, age, individual identity, and group affiliation of the calling individual [6–10]. While social calls are intentionally produced to transmit information to other individuals, echolocation calls usually unintentionally provide information to eavesdroppers [2, 5].

A striking behavioral pattern that is closely related to social group interactions in bats is swarming. It is characterized by intensive flight activity of several individuals in the vicinity of the roost. Swarming bats show typical flight behaviors like chasing flights and repeated approaches towards the roost entrance with short landings [3, 11, 12]. In many temperate bat species, swarming can be observed in spring and summer at maternity roosts when bats return from foraging and in autumn at swarming sites that are often near hibernacula [11–14]. Although there is a high similarity in the overall behavioral pattern between both situations, the social context differs between the two seasons. In temperate bat species, females gather in maternity colonies from early spring until summer to birth their pups. Bats benefit from roosting and breeding in groups because it facilitates mutual warming, cooperation, and safety from predators [15]. Some species switch maternity roosts almost every day, most likely to avoid predation, improve microclimate conditions, reduce distances to food patches and lower parasite pressure [16–19]. In addition to this, bats inevitably become separated from conspecifics as they fly rapidly through their environment during foraging. To obtain the benefits of group-living, bats have to signal the roost location to conspecifics. Therefore, many bat species use specific social calls, so-called contact calls, to recruit conspecifics to roost locations and maintain group cohesion [2]. Contact calls show individual-specific signature characteristics in different animal taxa, including bats [20–22]. Studies on Pallid bats (*Antrozous pallidus*) and Noctule bats (*Nyctalus noctula*) revealed that contact calls are frequently produced during dawn swarming [13, 21]. Furthermore, Schöner et al. [23] showed that Bechstein’s bats (*Myotis bechsteinii*) and Natterer’s bats (*Myotis nattereri*) are attracted to the social calls of conspecifics when they are seeking roost locations. Moreover, dawn swarming itself is proposed to function as an additional cue for individuals in the vicinity of the roost [12].

Intense swarming behavior also occurs in many temperate bat species at underground sites in late summer and autumn after maternity colonies have dispersed [11, 14, 24]. Autumn swarming is accompanied by intense social vocalizations that are associated primarily with mating [14, 25]. In most swarming bat species, males outnumber females at autumn swarming sites [14, 24, 26, 27]. Genetic studies revealed that autumn swarming increases genetic diversity within populations, which is especially important for highly philopatric bat species [26, 28, 29]. Many swarming species show promiscuous mating behavior [4], where males compete for female mating partners. Besides mating, autumn swarming may also play a role in assessing the suitability of a site as a hibernaculum [11, 14].

In this study, we focused on *M*. *nattereri*, which is widespread in Europe and one of the most common species at autumn swarming sites. Pfalzer [30] described four call types of *M*. *nattereri* that were recorded in a maternity roost as well as during swarming in the vicinity of the roost. Two of them were variable frequency modulated calls. Another one contained sequential steeply modulated FM-pulses. The last one began with a squawk-like noisy signal terminated in a tonal signal part. However, in-flight social calls of Natterer’s bats at autumn swarming sites have not yet been described and little is known about the function of social calls during swarming in different behavioral contexts. Maternity colonies of Natterer’s bats are found in cervices and tree cavities, and the roosts are frequently changed. Intensive dawn swarming can be seen especially immediately before and after the roost is changed [31]. On average, maternity colonies contain 20–100 females and a small number of males may also be present in the same roost [4]. Natterer’s bats show promiscuous mating behavior with mating predominantly occurring at autumn swarming sites, but opportunistic mating can also occur in the hibernaculum. As some males are also present in the maternity roosts, mating may also occur in the vicinity of the roost [4]. The swarming activity of *M*. *nattereri* can be observed at autumn swarming sites from mid-August until mid-November with peak activity from mid-September to mid-October [26].

Although the swarming behavior of Natterer’s bats has been the subject of former studies [14, 26] and different social calls of *M*. *nattereri* have been described [30], the swarming-related social calls have been barely investigated. The aim of this study was to record and describe the social call repertoire of free flying Natterer’s bats during swarming activity at the maternity roost and at the autumn swarming site. We hypothesized that social vocalizations differ between the two swarming sites and that the structure of the social calls mirrors the behavioral context. As swarming at the maternity roost is proposed to facilitate group cohesion, we expected a high abundance of short vocalizations that facilitate individual recognition. Autumn swarming occurs during the mating season. Therefore, we expect a high abundance of complex vocalizations that possibly function as mate attraction calls.

## Materials and methods

### Ethical statement

No specific permits were required for the described field studies since only sound recordings were made and no specimens were sampled and/or handled. No privately owned or protected land was accessed during the recordings. Field studies did not disturb endangered or protected species.

### Study site and species identification

The swarming behavior of free-flying Natterer’s bats was studied at one maternity roost and one autumn swarming site in the Neckar-Alb region in South-West Germany. The studied maternity roost was located near Tübingen at “Kloster Bebenhausen” (48°33′ N lat., 9°03′ E long.). The recording period at the maternity colony lasted from early July to late August. During this period, we recorded the swarming activity of *M*. *nattereri* for ten nights. Recordings were conducted from 1.5 hours before sunrise until no more flight activity was observed. During that time window, the highest swarming activity was expected. Most of the time the bats were roosting in a loose sandstone wall about 5 meters above the ground, but the roost location changed three times during the recording period. As bats entered the roost after they were swarming, the chance to record an individual disproportionally often on one night was low. Furthermore, single individuals of this species tend to switch their roost frequently [19], which also reduces the risk of pseudoreplication. To investigate the autumn swarming behavior of *M*. *nattereri*, we conducted recordings at an underground site at the northern edge of the Swabian Alps known as “Dettinger Höllochschacht” (48°33′ N lat., 9°20′ E long.). The cave entrance has a diameter of approximately 1 meter and is protected by a grate. There, we recorded the swarming activity during two nights at the end of September. The recordings were conducted from 11 pm to 1 am when the highest swarming activity was expected. During both recording sessions a large number of individuals were swarming simultaneously. Often, single individuals entered the cave or flew out of the observed area. Thus, the chance of recording the same individual disproportionally often was low.

We identified the different bat species by their echolocation signals. The echolocation calls of *M*. *nattereri* are characterized by their steep, almost linear frequency modulated (FM) signal, with an average initial frequency of 135 kHz and an average terminal frequency of 16 kHz [32]. Social calls were attributed to a species according to the frequency structure, and rhythm and intensity of adjacent echolocation calls.

### Data recordings

Sound recordings were conducted using a unidirectional custom-made ultrasonic microphone (Animal Physiology, University of Tübingen). The analog signals were amplified (20 dB), digitalized (sampling rate: 384 kHz, 16-bit) and stored as wav-files with PC-Tape (Animal Physiology, University of Tübingen). We used a portable recording setup with post trigger to localize roosts and areas of high swarming activity. Afterwards swarming was recorded with a stationary ultrasonic microphone and an IR camera (Sanyo, IR CCD, Japan) recording at a rate of 50 Hz, both connected to PC-Tape, which allowed the synchronization of sound and video recordings. Each half frame was illuminated for 1 ms using a custom-made LED infrared flash (Animal Physiology, University of Tübingen). The videos were stored on Panasonic DVC mini-tapes using a digital video camera recorder (Sony, DCR-TR V30E, Japan). At the maternity roost, the microphone was positioned directly beneath the roosting crevice and was orientated away from the wall in an upward direction 45° off the horizontal position. The camera was focused on the microphone. At the autumn swarming site, the microphone was positioned 1 m above the ground of the ravine, 3 m from the cave entrance pointing upwards. The camera was installed at an elevated position and was focused on the area above the cave entrance.

### Analysis of social vocalizations

The recordings were displayed as color spectrograms (FFT 256, Blackman window, dynamic range 80 dB) with custom-written Selena software (Animal Physiology, University of Tübingen). To analyze the recordings, signals were displayed in a 512×512-pixel precise window, with frequency range of 0–180 kHz, and duration of 40 ms. Through auto padding and overlap of the spectra, a resolution of Δf = 350 Hz and Δt = 0.08 ms were achieved.

Social calls were classified by call type according to their signal structure in the spectrogram and the measurements of different call parameters. The nomenclature of call structure followed that of Middleton et al. [4]. We used various parameters to describe and analyze social calls (Table 1). The beginning and end of a call was set at -30 dB relative to the maximum amplitude of a respective call. To reduce pseudoreplication we never used consecutive social calls for general call structure analysis and only analyzed three social calls of the same call type from two minutes of recording. Echolocation calls were analyzed based on the same criteria as social calls. We performed a two-way ANOVA with the factors call type and swarming site to determine if the calls that had been assigned to the same call type by their signal structure differed between the recording sites. In two-element social calls each element was analyzed as individual call.

**Table 1:** Description of parameters measured from social calls in swarming Natterer’s bats.

General call parameters (type A–D):	Description
Call duration	Time span between beginning and end of a call
Bandwidth	Total frequency range that is covered by a call
Initial frequency (F_start_)	Frequency at the beginning of a call
Terminal frequency (F_end_)	Frequency at the end of a call
**Call type A specific parameters**:	
Duration a to b (Dur_ab_)	Time span between point a and b
Duration b to c (Dur_bc_)	Time span between point b and c
Frequency b (F_b_)	Frequency at point b
Frequency c (F_c_)	Frequency at point c
**Call type D specific parameters**:	
Maximal frequency (F_max_)	Highest frequency of a call
Minimal frequency (F_min_)	Lowest frequency of a call
Local minimal frequency (LMF)	Lowest frequency within one oscillation of a trill
Modulation frequency	Reciprocal of the mean period duration of a trill
Mean bandwidth	Frequency range that is covered within one period

Furthermore, we investigated whether call type A showed an individual-specific signature. We analyzed 15 different sequences chosen from six different nights to minimize the chance of pseudoreplication. From each sequence we measured three consecutive calls of type A that were unambiguously assigned to a single individual according to the rhythm and intensity of previous and subsequent echolocation calls. For each call, we measured the start frequency (point a), frequency of the turning point from the shallowly modulated to the steeply modulated component (point b), and terminal frequency (point c). A linear discriminant function analysis (DFA) was conducted on four, minimally correlated acoustic parameters: Dur_ab_, Dur_bc_, F_b_ and F_c_. Using these parameters, the DFA generates discriminant functions that are correlated with the original values. The influence of each parameter on each discriminant function can be determined via the canonical coefficients. The classification success for each call to an individual was cross-validated using the “leave-one-out” method. In this method, each call was classified using the discriminant functions derived from all other calls except the one that should be classified. This process was repeated until all calls had been left out once and had subsequently been classified. The overall significance of the models was estimated by comparing the classification success of the cross-validation method to the classification success expected by chance. A similar analysis of individual signatures was not possible for call types B-D as we did not record a sufficient amount of sequences that allowed an unambiguous assignment of multiple social calls to the same individual.

Further, we investigated the variability of type D calls in more detail. Vocalizations produced in the context of mating usually encode the fitness of the calling individual. If a call parameter is varied independent from other parameters, it might indicate the quality of the sender. Therefore, we performed covariance analysis using a correlation matrix on call duration, modulation frequency and mean bandwidth.

We compared the distribution of call types between both sites using a two-sample χ^2^ test conducted on 554 social calls from the maternity roost and 346 social calls from the autumn swarming site. For this analysis, we only used files that contained social calls from different nights, in total summing up to 45 minutes of sound recordings for each site. The total number of each call type was counted and set in relation to the total number of social calls produced.

Statistical analysis was performed using Microsoft Excel 2013 and JMP v13.0.0 (SAS Institute Inc., USA). Nonparametric tests and the DFA were carried out using IBM SPSS Statistics v22 (IBM Corporation, USA). For all statistical tests, the significance level was set to α = 0.05.

## Results

### Behavior during swarming activity

At the maternity roost, swarming activity started approximately one hour before sunrise. In some cases, individuals were present in the vicinity of the roost even earlier. Typically, bats swarmed in small groups of two to four individuals for a few minutes before they entered the roost in quick succession. After a pause of another few minutes, the next group of bats appeared and began to swarm. Bats performed circular flights and passed the roost entrance frequently. Often, two or more individuals performed chasing flights that were accompanied by social vocalization. Furthermore, we frequently observed hovering flights immediately in front of the crevice, occasionally accompanied by short landings on the wall. Some individuals exited the roost again, which was often accompanied by the emission of social calls.

At the autumn swarming site, bats showed high swarming activity during the entire period of observation. Three bat species were recorded at the autumn swarming site, which were clearly distinguishable from their echolocation calls with *M*. *nattereri* being the most common species at this site, followed by *Myotis myotis*, and *Pipistrellus pipistrellus*. Usually, more than five individuals swarmed simultaneously. Individuals performed circular flights in the shallow ravine in front of the entrance, which lasted for several minutes. Additionally, bats performed chasing flights with up to four individuals following each other. The highest activity was observed in close proximity to the cave entrance. There, bats often hovered in front of the grate, which coincided with intense social vocalization. In some cases, we observed single individuals performing stereotypical oscillating flights along the grate, which were also associated with social vocalization.

### Social call repertoire

**Fig 1:**
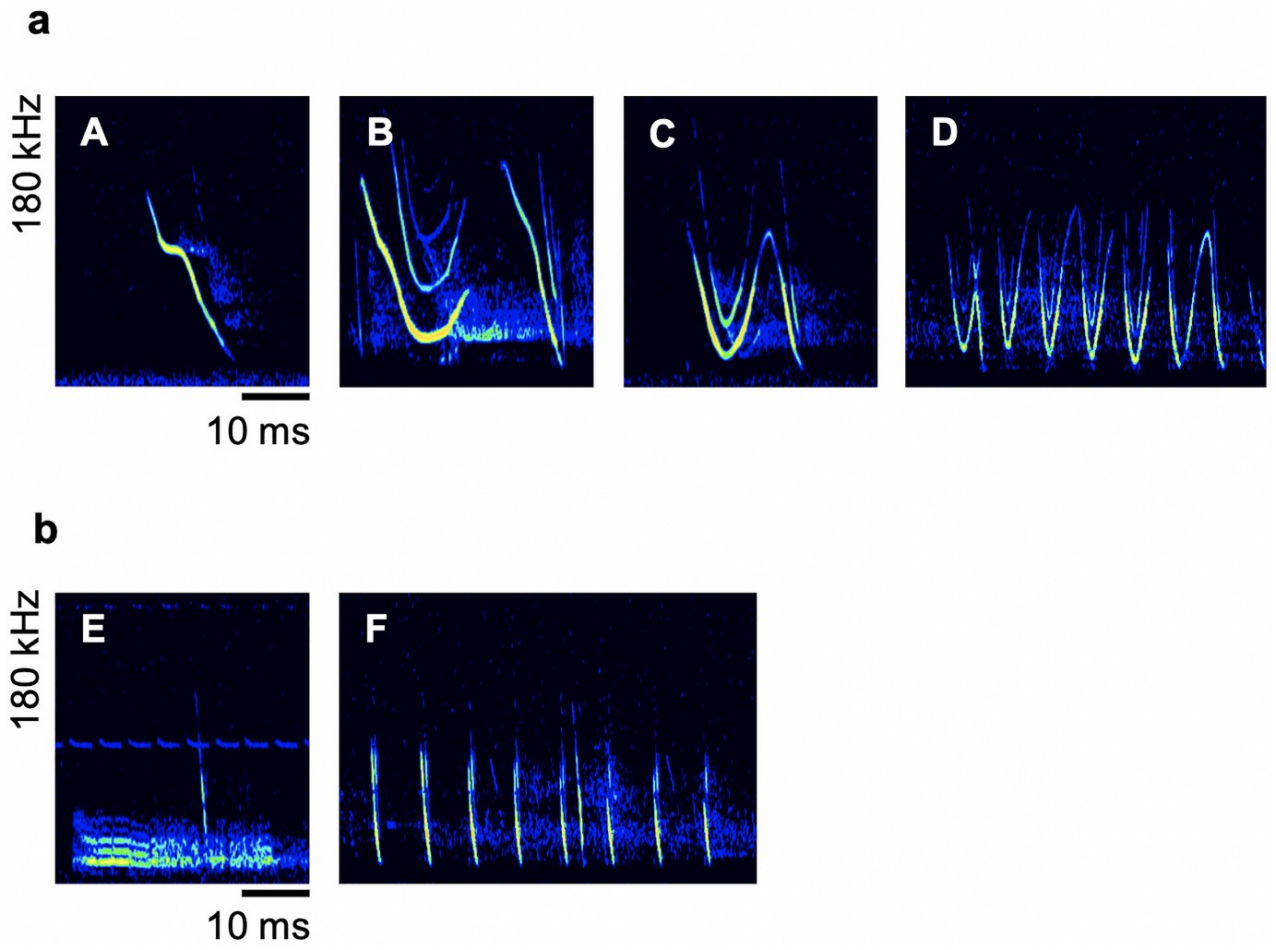
Spectrograms of six call types of the social call repertoire of swarming *Myotis nattereri*. **a** Commonly recorded call types. (A) Short cheep-like call characterized by a steep frequency modulation with a shallowly modulated middle part. (B) Two element call with an upward hooked element followed by a steep frequency modulated (FM) element. The second element was omitted occasionally. (C) Fast modulated call characterized by a rapid downward-upward-downward frequency modulation. (D) Long broadband trill. **b** Rarely recorded call types. (E) Squawk with noisy signal. (F) Churring-like call consisting of short FM pulses.

The social call repertoire of swarming Natterer’s bats consisted of six different call types that showed a characteristic signal structure, which allowed an unambiguous discrimination between call types. Four of them were very common (Fig 1a and S1 Fig) whereas two of them occurred very rarely (Fig 1b). Due to their comparatively high abundance, we assumed that calls of types A-D (Fig 1a) are the most important call types for communication in swarming Natterer’s bats. Thus, we only qualitatively describe call type E and F and focused exclusively on the four common call types in further analyses.

All four common call types showed a variable frequency modulated signal structure (Fig 1a). We analyzed the two call parameters call duration and bandwidth for all social calls (Table 2) and compared them between the two swarming sites. As expected, the call type had a significant effect on call duration (F(4,160) = 122.99, P<0.0001) and bandwidth (F(4,160) = 6.93, P<0.0001) and the swarming site had no significant influence on call duration (F(1,160) = 0.52, P = 0.4736) or bandwidth (F(1,160) = 0.20, P = 0.6590). There was an interaction between call type and swarming site for call duration (F(4,160) = 2.86, P = 0.0251) and bandwidth (F(4,160) = 3.35, P = 0.0115). The interaction of call type and swarming site on the call duration is mainly influenced by the differences in call type D. Calls of this type were longer at the autumn swarming site. We conducted post hoc orthogonal t-tests for the call duration of the different call types. There were no differences between the two swarming sites (P>0.05) except call type D (P = 0.0037). The interaction of call type and swarming site on the bandwidth was influenced by call type B, C and D. Both elements of type B had a smaller bandwidth at the autumn swarming site whereas call type C and D had a slightly higher bandwidth at the autumn swarming site. These differences were not significant (orthogonal t-test, P>0.05) except for the first element of call type B (orthogonal t-test, P = 0.0015).

**Table 2:** Mean and standard deviation of call parameters of echolocation calls and the four common call types. The range is given in parentheses.

Call type	Element	Call duration [ms]	Bandwidth [kHz]	F_start_ [kHz]	F_end_ [kHz]
**Echolocation** (n = 40)	-	3.3 ± 0.8 (2.0–5.0)	125.4 ± 9.6 (103.3–142.4)	141.1 ± 8.4 (119.5–155.2)	15.7 ± 4.0 (32.3–11.6)
**A** (n = 50)	-	9.5 ± 1.8 (5.7–14.5)	96.6 ± 11.1 (69.6–118.6)	113.2 ± 11.1 (88.7–132.7)	16.5 ± 5.5 (11.2–44.0)
**B** (n = 32)	Total	28.4 ± 5.0 (19.4–37.1)	–	110.6 ± 12.4 (89.2–134.4)	15.9 ± 4.2 (9.7–25.8)
	1	13.6 ± 2.3 (8.5–17.0)	88.5 ± 16.1 (116.0–54.3)	110.6 ± 12.4 (89.2–134.4)	48.4 ± 19.1 (22.5–94.8)
	2	7.2 ± 1.6 (4.5–10.2)	103.8 ± 14.3 (78.7–130.6)	119.8 ± 15.1 (91.0–146.5)	15.9 ± 4.2 (9.7–25.8)
**C** (n = 34)	-	19.5 ± 2.1 (16.5–24.7)	85.5 ± 13.8 (59.0–112.7)	101.9 ± 14.1 (75.2–130.9)	16.4 ± 4.0 (12.0–25.0)
			**Mean Bandwidth [kHz]**	**F**_**max**_ **[kHz]**	**F**_**min**_ **[kHz]**
**D** (n = 22)	-	35.8 ± 11.1 (22.4–65.1)	74.8 ± 9.4 (58.8–92.5)	113.2 ± 11.3 (90.2–139.4)	16.0 ± 5.3 (10.2–28.5)

Calls of type A (Fig 1a and S2 Fig) showed an individual signature. Based on the four chosen call specific parameters, the DFA classified 18 of the 45 (40.0%) type A calls correctly to different individuals. Random classification would result in a classification accuracy of 6.67%. The first three discriminant functions had a significant discriminating power and described 49.6%, 27.9%, and 13.6% of the inter-individual variability, respectively.

**Fig 2:**
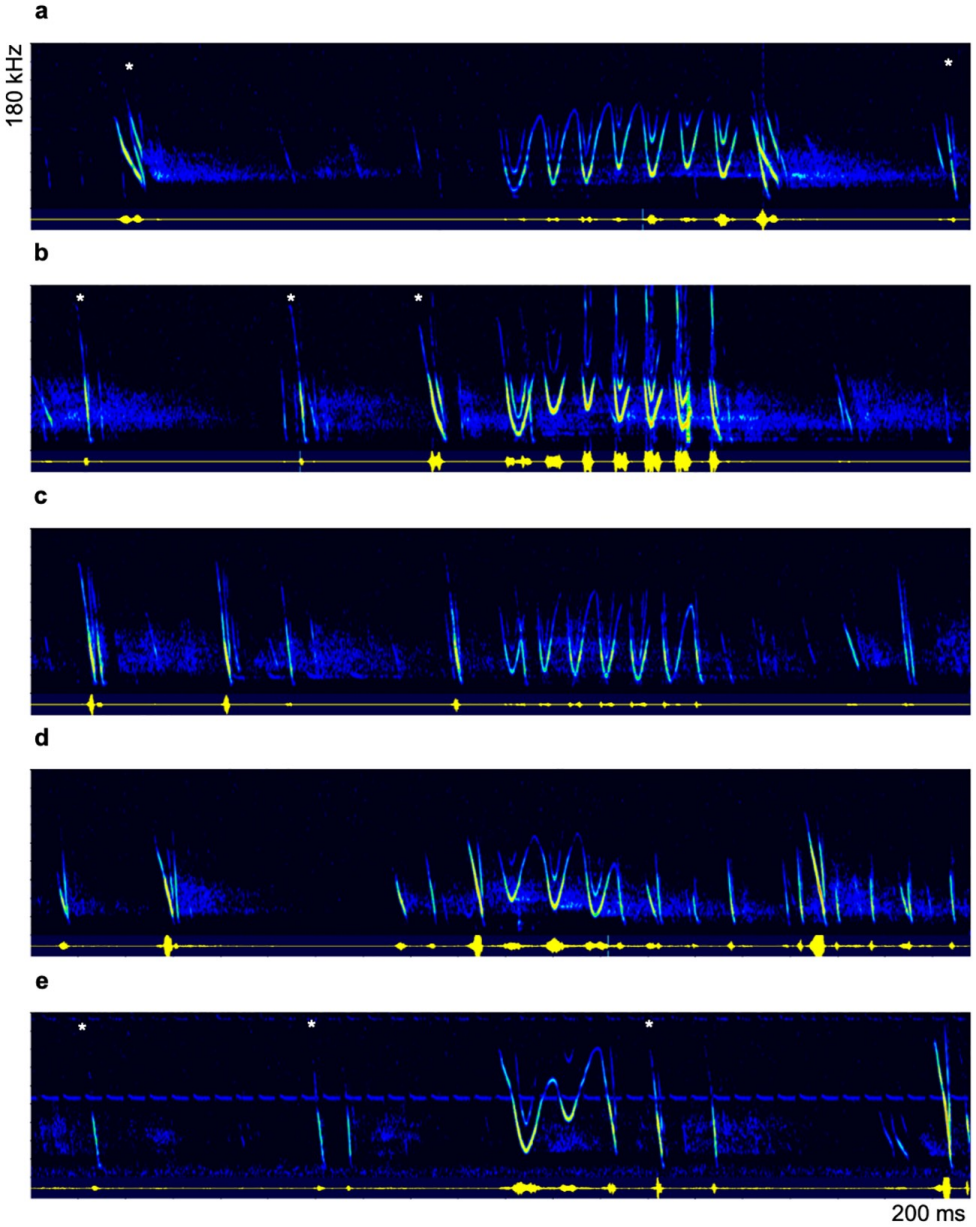
Variability of call type D. Exemplary spectrograms of five different calls of type D showing the high variability of this call type. Social calls are produced in flight and are integrated between echolocation signals. Echolocation calls of the respective individual are marked with asterisk (_*_) if an unambiguous assignment was possible.

Call type D (Fig 1a) differed from the other call types due to its long duration and characteristic sinusoidal trill-like signal structure. These calls exhibited a high variability (Fig 2). The bandwidth was approximately 75 kHz, but the initial and terminal frequency varied over a wide range. Additionally, the modulation frequency differed between the calls, with the lowest modulation frequency at 101 Hz and the highest modulation frequency at 151 Hz. The changes in the local minimal frequency (LMF) over time showed no uniformity between calls. In some calls, the LMF was kept relatively constant (e.g., Fig 2c), whereas some calls showed strong variations in the LMF during the progression of the call (e.g., Fig 2e). Furthermore, the upper half of the trill was missing in some calls whereas the lower frequencies were clearly pronounced (e.g., Fig 2b). Three parameters that strongly contribute to the high call variability were found to be independent of each other. The call duration had no influence on the mean bandwidth or modulation frequency (r^2^ (22, 22) ≤ 0.06; P ≥ 0.2827) and the modulation frequency had no influence on the mean bandwidth (r^2^ (22) = 0.11; P = 0.1368).

### Distribution of different call types at the two swarming sites

**Fig 3:**
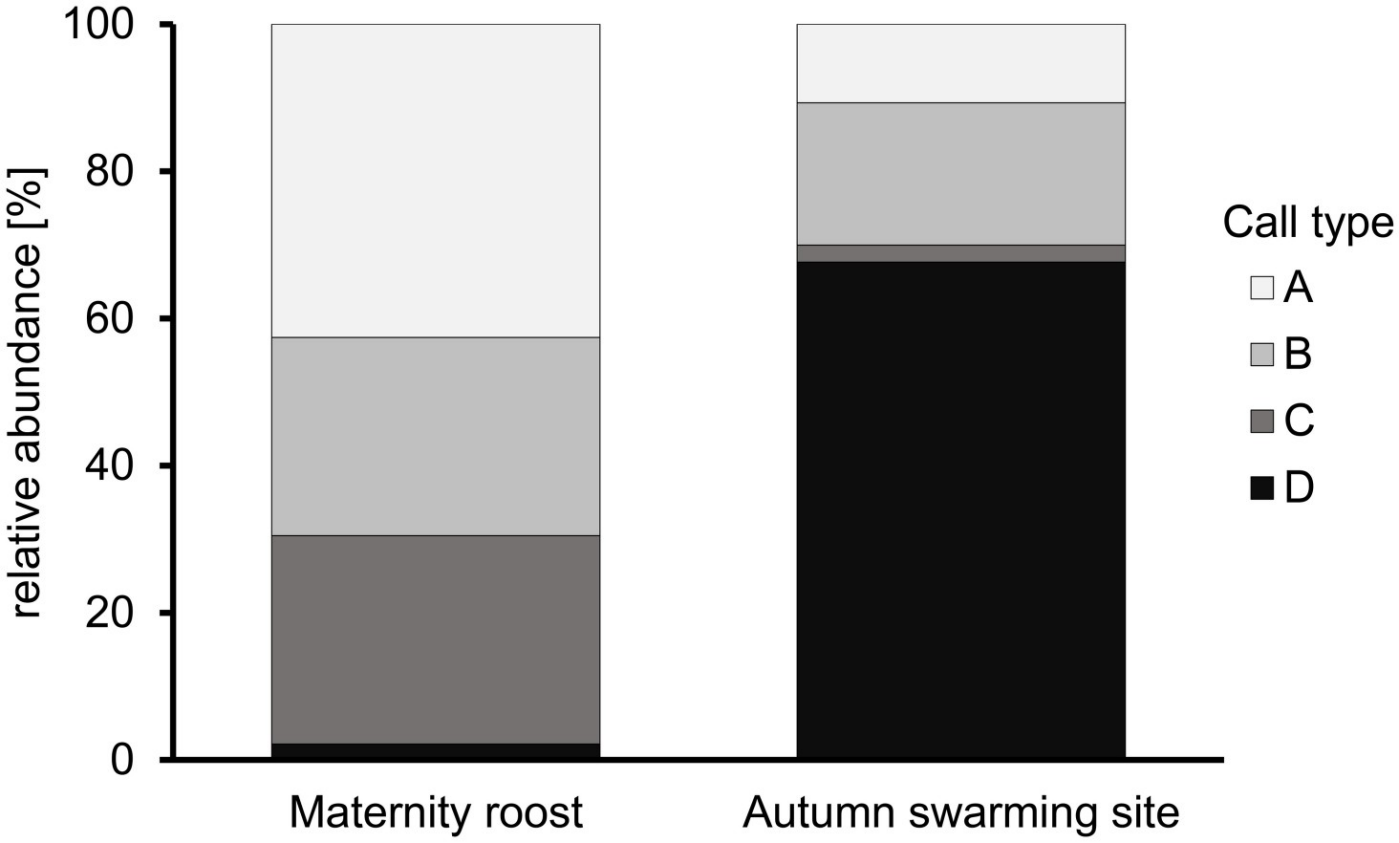
Distribution of different call types. Comparison of the relative abundance of the commonly recorded call types at the maternity roost and autumn swarming site.

The distribution of call types clearly differed between the maternity roost and autumn swarming site (χ^2^ (3) = 489.14; P < 0.0001) (Fig 3). At the maternity roost, call type A (42.6%) was the most common social call, followed by call type C (28.3%) and call type B (26.9%). However, call type D (2.2%) was recorded very rarely during swarming at the maternity roost. It only occurred in two sequences in late July and late August. From the integration of the trills in the course of the echolocation signals, it can be assumed that in both situations the trills were emitted by a single individual. At the autumn swarming site, the distribution of call types showed a completely different pattern. Here, call type D (67.6%) clearly dominated the entirety of emitted social calls. Call type A (10.7%) and call type B (19.4%) were much less common and call type C (2.3%) almost never occurred at the autumn swarming site.

## Discussion

### Swarming behavior and vocal repertoire

Natterer’s bats showed high swarming activity at the maternity roost, as well as at the autumn swarming site. At both sites they exhibited flight behaviors that are typical for bat swarming [3, 11, 30], like chasing flights and repeated approaches to the roost entrance with short landings. Swarming was always associated with social vocalizations. We classified the social call repertoire into six call types. Social calls of *M*. *nattereri*, which are similar to call types B, C, E, and F, have already been described in previous studies [30]. However, to our knowledge, call types A and D have not yet been described.

Pfalzer [30] described a call similar to call type C as a “V-shaped” social call followed by an FM element. Due to the signal structure and similarity of the call parameters, we assume that this call type is equivalent to call type C in the present study. The missing connection between the V-shape element and subsequent FM part in Pfalzer’s study may be a recording artefact due to a low signal-to-noise ratio. Furthermore, Pfalzer [30] described a social call that is similar to the first element of call type B in the present study. As we also recorded the first element solely from time to time and all other parameters coincide, we assume that both descriptions refer to the same type of social call. Pfalzer [30] also recorded both calls during high swarming activity at the maternity roost just before sunrise. Social calls similar to call type E and F are also reported to be uttered by Natterer’s bat during swarming at the maternity roost [30]. Based on its signal structure, call type E can be classified as a squawk call, a type that is typically uttered during agonistic interactions and are found in many other bat species [3, 33, 34]. Call type F is also described by Pfalzer [30]. Due to its signal structure, this call is considered to be a distress call that is typically uttered during increased irritation [3, 5].

### Differences in the relative abundance of call types at the two swarming sites

Bats swarming at the maternity roost are in a different social and behavioral context than bats swarming at the autumn swarming site. Thus, we expected explicit differences in social vocalization behavior. Although, the fundamental vocal repertoire of *M*. *nattereri* did not differ between the two sites, we found a distinct difference in the relative abundance of call types. In general, social calls uttered in a specific situation encode for information that plays an important role in this social behavioral context. Particularly in bat species, that frequently change roost sites, swarming activity in combination with social vocalization is supposed to increase group cohesion and facilitate roost formation [12, 21]. Calls of type A, B, and C were common at the maternity roost; thus, short tonal social calls seem to play the most important role for Natterer’s bats in this context. In contrast to this, trills of call type D seem to transmit information that is not important during swarming at the maternity roost but is important at the autumn swarming sites. At the same time, rather short social calls were uttered less frequently and seem to be less relevant in this social context. Sex-specific calls may play a role as usually, more females than males are present during swarming at maternity roosts whereas a strongly male-biased sex ratio has been reported in studies involving autumn swarming [14, 26, 27]. Thus, it is possible that social calls that are typically produced by females are overrepresented at maternity roosts and vice versa, social calls that are typically produced by males are overrepresented at autumn swarming sites.

### Short social calls may function as contact call

Different studies addressing swarming behavior at maternity roosts and the corresponding vocalizations indicate that that they function as beacons to potential roost mates to the roost location and that swarming is found especially in bat species that switch the roosts frequently [12, 13, 21, 23]. Arnold and Wilkinson [21] reported that short contact calls are associated with dawn swarming activity in *A*. *pallidus*. We assume that calls of type A, which were the most frequently uttered social call during swarming at the maternity roost, functions as contact calls in *M*. *nattereri*. The DFA revealed individuality in type A calls, a typical trade of contact calls [2]. Although, we analyzed only three calls of each individual and used four rather conservative parameters, the function classified significantly more calls correctly to an individual than expected just by chance. We are aware that the similarity of the calls may be biased by our method of using three consecutive social calls to assign single social calls to the same individual. The similarity between consecutive calls may generally be higher than between calls of the same individual chosen from different sequences.

As call type B and C were also frequently uttered during dawn swarming, they may also be used by bats to recruit conspecifics. The abundance of call type B showed the least difference between both sites; thus, it seems to be important in both social contexts. Call type C has a function that is important at the maternity roost but not at the autumn swarming site. Playback studies with Natterer’s bats, using social calls similar to call type C, revealed that single individuals were more attracted by this social call than by heterospecific social calls when searching for new roosts [23].

### Long trills most likely function as advertisement calls

The trill-like type D calls showed the most salient signal structure within the recorded vocal repertoire of swarming Natterer’s bats. Trills almost never occurred during pup rearing at the maternity roost but were very common at the autumn swarming site. Hence, they most likely fulfill an essential function during autumn swarming and seem to be closely linked to the mating behavior of Natterer’s bats. Like in other autumn swarming bat species, the mating system of *M*. *nattereri* is described as promiscuous, where females choose the most competitive male for mating [4]. Different studies addressing autumn swarming, including in Natterer’s bats, reported a male-biased sex ratio at autumn swarming sites [14, 24, 26, 27] and the high vocalization activity of males at autumn swarming sites has been suggested to attract female mating partners [25]. The proposed advertisement function of trills is supported by the fact that type D calls occurred only very rarely at the maternity roost during breeding season. Furthermore, type D calls were significantly longer at the autumn swarming site which fits to the proposed signal design of social calls in a mating context [5, 35]. All trills at the maternity roost were recorded in late July and August. As males are also present in the maternity roost in small numbers [4], some individuals may have already started their displays.

Vocal displays are documented in more than 20 bat species and are especially well studied in the *Pipistrellus* genus and in some tropical bat species [3, 36–39]. Trill-like vocalizations are associated with mating in several of these species. In *Saccopteryx bilineata*, male songs function as acoustic beacons for female individuals [40]. This species shows a multimodal courtship behavior, where males utter complex songs for courtship [41]. The most common element of these songs are trills, which show significant differences between individuals [37]. Trill calls also play an important role in the male courtship displays of *Carollia perspicillata* [39]. It has been suggested that male vocalization transfers information about the quality of the sender and that individuals with a higher number of unique elements in their vocal repertoire have higher reproductive success [42]. Another species that exhibits a rich vocal display behavior is *Tadarida brasiliesis*. During the mating season, males produce elaborated songs to defend sites where reproductive females are roosting. These songs contain trill elements that are produced by males that are present at the roost site [5].

Due to the high similarity in signal structure and behavioral context to other bat species, we propose that trill-like social calls are uttered by male Natterer’s bats and function as advertisement calls to attract females for mating. The comparatively complex signal structure of the described trills fits the theoretical design features of advertisement calls [5, 35]. Trills were highly variable in call duration, modulation frequency, and modulation range, and these parameters changed independently from each other within and between calls. Consequently, these parameters may differ among males depending on the vocalization abilities of the calling individual. Trills potentially indicate the quality of a male individual and specific features of trill calls could function as honest signals for female Natterer’s bats.

Non-acoustic cues may also influence the reproduction success of male Natterer’s bats. Several bat species, such as *Pipistrellus nathusii* [43], perform stereotypic flight behaviors during courtship that may play a crucial role in mating [37, 39]. *M*. *nattereri* individuals also exhibited various conspicuous flight behaviors at the autumn swarming site, which may be part of a complex display behavior that indicates the quality of potential mating partners.

In conclusion, we revealed six swarming related social calls of *M*. *nattereri*, of which two are described for the first time in the present study. One of them, call type A, has an individual signature and most likely supports group cohesion during the breeding season. The other one, call type D, is highly variable and most likely an advertisement call that is closely linked to the mating behavior of Natterer’s bats. To fully understand the function of social calls in bats it is necessary to study social communication in its behavioral context.

## Supporting information

S1 Fig Social calls in echolocation context. Exemplary spectrograms of the four common social calls (SC). Social calls are produced in flight and are integrated between echolocation signals (_*_).
(TIF)

S2 Fig Individuality in call type A. Exemplary spectrograms of call type A from four different individuals.
(TIF)
