# Supplementary figures and images for: Social calls of Myotis nattereri during swarming: Call structure mirrors the different behavioral context
Source: PLoS One. 2019 Sep 6;14(9):e0221792. doi: 10.1371/journal.pone.0221792 (PMC6730923; doi:10.1371/journal.pone.0221792)

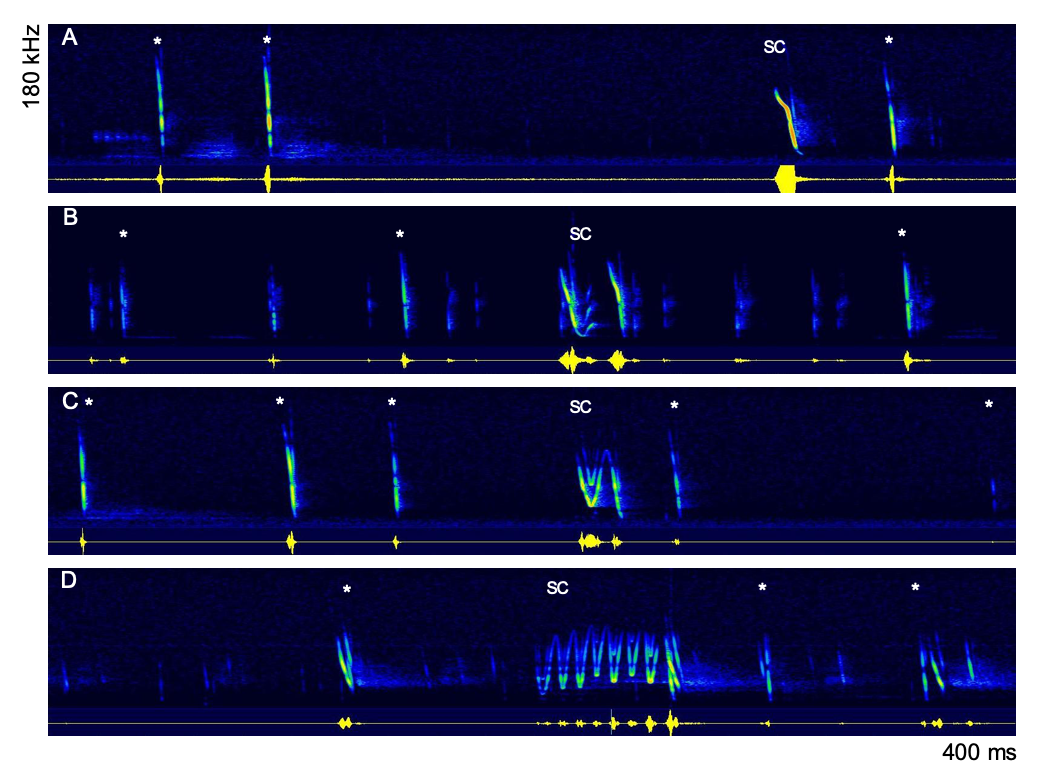

Supplement: S1 Fig — Exemplary spectrograms of the four common social calls (SC). Social calls are produced in flight and are integrated between echolocation signals (*). (TIF) [file pone.0221792.s001.tif]

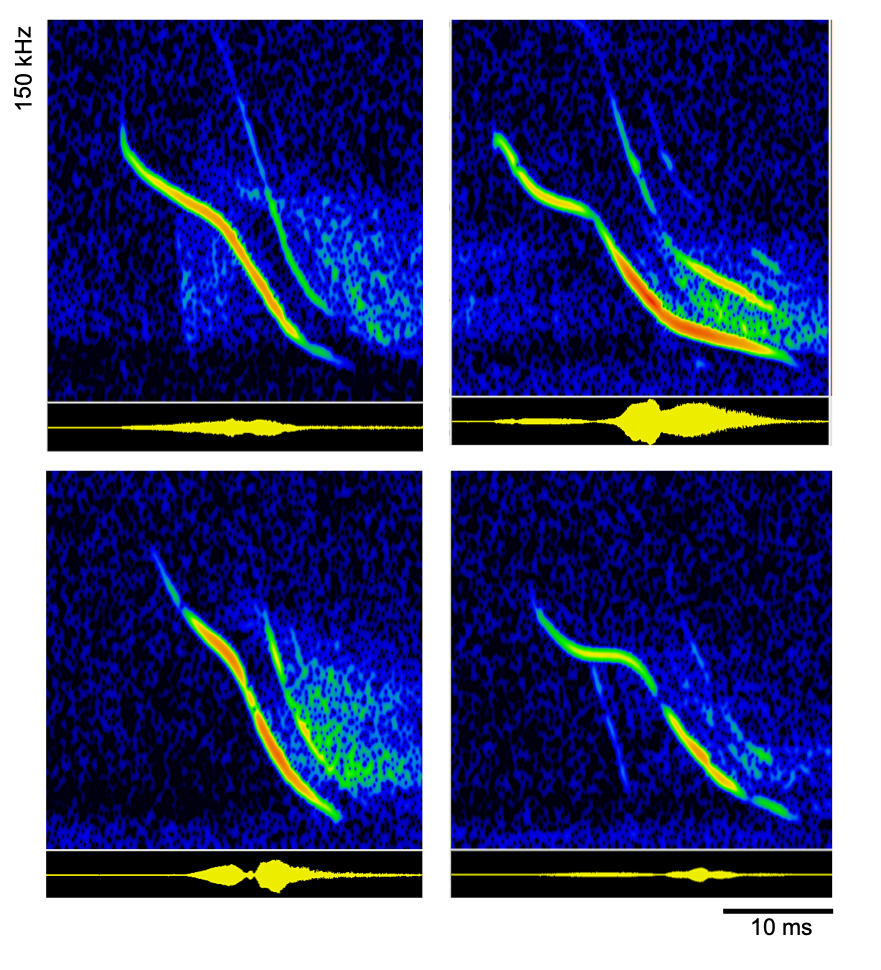

Supplement: S2 Fig — Exemplary spectrograms of call type A from four different individuals. (TIF) [file pone.0221792.s002.tif]
